# Heterophyllous Shoots of Japanese Larch Trees: The Seasonal and Yearly Variation in CO_2_ Assimilation Capacity of the Canopy Top with Changing Environment

**DOI:** 10.3390/plants9101278

**Published:** 2020-09-28

**Authors:** Satoshi Kitaoka, Qu Laiye, Yoko Watanabe, Makoto Watanabe, Toshihiro Watanabe, Takayoshi Koike

**Affiliations:** 1 Faculty of Earth Environmental Sciences, Hokkaido University, Sapporo 060-0810, Japan; skitaoka3104@gmail.com; 2 Research Center for Eco-Environmental Sciences, Chinese Academy of Sciences, Beijing 100085, China; 3 Research Facultyof Agriculture, Hokkaido University, Sapporo 060-8589, Japan; youko@for.agr.hokudai.ac.jp (Y.W.); nab0602@cc.tuat.ac.jp (M.W.); nabe@chem.agr.hokudai.ac.jp (T.W.); 4 Institute of Agriculture, Tokyo University of Agriculture & Technology, Tokyo 183-8509, Japan

**Keywords:** Japanese larch (*Larix kaempferi*), P_sat_–N relation, V_cmax_–N, J_max_–N, heterophyllous shoots, nitrogen remobilization rate, year-to-year variation

## Abstract

Japanese larch (*Larix kaempferi* = *L. leptolepis*) is often characterized by its high growth rate with heterophyllous shoots, but the functional differences of heterophyllous shoots still remain unclear. Recently, abrupt high temperature and drought during spring induced high photosynthetic rate via change in leaf morphology of the deciduous habit. In order to reveal the photosynthetic characteristics of both short and long-shoot needles of sunny canopy of the larch trees using a canopy tower, we calculated the seasonal change of gas exchange characters and leaf mass per area (LMA) and foliar nitrogen content (N) of heterophyllous needles: short and long-shoot needles over 3 years. No marked difference in light-saturated photosynthetic rates (P_sat_) was observed between short and long shoots after leaf maturation to yellowing, although the difference was obvious in a specific year, which only shows that seasonal change in temperature and soil moisture determines the in situ photosynthetic capacity of needles. The large annual and seasonal variations in P_sat_ in both shoots were found to be mainly determined by climatic variations, while shoot types determined the strategy of their photosynthetic N utilization as well as the stomatal regulation.

## 1. Introduction

Plants have high morphological and functional plasticity because they do not have the ability to move in order to avoid harsh environments and conditions [1,2]. According to the Fifth Assessment Report of IPCC [3], annual fluctuation in terms of climate condition has been observed to increase in the past years. As a result, we recently have extremely warm weather during spring with little precipitation [4,5]. The photosynthetic rate of some deciduous tree seedlings responds well to warm springs or drought in terms of leaf morphological changes and leaf nitrogen (N) accumulation [6,7,8]. Moreover, under stress conditions (light, water, nutrient, etc.), plants can cope by changing their leaf mass per area (LMA) for efficient use of their resources as well as for allocation of biomass (e.g., [7,9]). The different roles of heterophyllous growth traits have been examined in birch [10,11,12], larch [13,14,15,16], and others [17,18].

Most larch (*Larix* sp.) species are characterized by its high growth rate with heterophyllous shoots [11,13] in deciduous leaf habit among conifer species [13]. Dahurian larch (*Larix gmelinii*) can grow on severe environmental conditions in East Siberia and Far East Russia, i.e., permafrost with small amounts of precipitation, extreme sunlight, huge daily temperature differences, etc. [19,20,21]. In addition, larch can survive at harsh environments but with mostly short-shoot needles, except in the initial stage of seedlings. Many studies have been carried out to determine the environmental responses of larch seedlings for long-shoot needles but not many on adult trees for short- and long-shoot needles [22].

We have been fascinated with the high growth rate of larch (*Larix* sp.) species and aimed to analyze their photosynthetic function [14,23,24,25,26,27]. Irrespective of the former expectation, it was concluded that no special metabolic pathway could be found [28], but the high photosynthetic rate may be realized through a unique arrangement of the different types of needles in a larch canopy (i.e., the heterophyllicity) [7,16,24] and deciduous leaf habit for avoiding cold and dry season and re-accumulation of N to plant body [25,29]. Is there any specific functional difference in heterophyllous shoots, i.e., short and long shoots of the Japanese larch? As deciduous leaf habit [2], we should determine the remobilization capacity of N before leaf shedding for the next growing season.

The roles of heterophyllous leaves were reported in birch, which has similar photosynthetic traits to larch [20,29], namely, early leaves and late leaves. The growth of late birch leaves was substantially suppressed when shading occured for early leaves [12,16]. In Japanese larch, the temporal growth patterns of the apices of short and long shoots were found to differ: the apices of long shoots have continual growth phases, but short shoots do not repeat annually the formation of winter buds [30]. Therefore, the growth of larch is strongly dependent on the photosynthetic activities of short-shoot needles. We expected that short-shoot needles should have high photosynthetic rate under various environmental conditions.

However, there are two contradictory results regarding the photosynthetic rate at light saturation (P_sat_) and ambient CO_2_ in Japanese larch, namely, short-shoot needles have lower P_sat_ than long-shoot needles [15]; in contrast, no difference in P_sat_ was found for both shoot types [14]. The defoliation treatment on short-shoot needles at lower canopy of Dahurian larch concluded that there is substantial suppression of both diameter and length growth at the upper canopy, though only the diameter is significantly decreased at the lower canopy [31]. These studies found that the functional role of these two shoot types may be different in crown development.

To assess the functional role of heterophyllous larch needles, in situ measurements should be performed, such as canopy photosynthesis in larches, including its hybrid [14,24,25,26], which is considered essential in understanding the carbon balance of a forest from leaf to stand [19,32,33,34]. However, studies examining the functional difference in heterophyllous larch shoot in situ are still very limited [22], and some of them were only good for one season’s results. There is a big yearly variation in P_sat_ among the four tree saplings in larch forests found in northern Japan, which is caused by the difference in air temperature and precipitation during leaf unfolding period [7,8,19,35]. If spring would bring few precipitation and high temperature, photosynthetic rate was high with high N, and this trend could be induced by manipulated experiments in a greenhouse [7]. Therefore, long-term measurement in situ will be needed to reveal the role and functional difference in heterophyllous shoots of larch species under certain field conditions.

As a typical heterophyllous conifer species, short shoots grow on older branches with bundles of needles, whereas long shoots of the Japanese larch generally develop at the top branches with separated needles directly on current-year branches [14,30]. However, the difference between short and long shoots has not well been discussed [16,20,26], and some ecophysiological questions still remain, e.g., differences in the relations between leaf N and photosynthetic rate (P_sat_), maximum carboxylation rate (V_cmax_) and maximum electron transportation rate (J_max_), and N remobilization rate before needle shedding [9,17,32]. Are there any variations in the relations of P_sat_–2N, V_cmax_–N, and J_max_–N between short- and long-shoot needles?

Photosynthetic capacity is substantially affected by the anatomical structure of leaves [36,37,38,39,40] as well as stomatal and mesophyll resistance in gas diffusion of water and CO_2_ [39,41,42]. Leaf functional structures, including leaf thickness and water availability, affect gas diffusion from air to chloroplasts and vice versa [39,40]. These former evidences are applicable to heterophyllous shoots in Japanese larch trees.

To address these questions, we monitored the in situ seasonal and annual changes of canopy photosynthetic capacity (P_sat_ and P_max_) in the needle traits of short and long shoots, together with environmental factors, using the canopy tower, from 2001 to 2003. The needle nitrogen remobilization rate (NRMA) and photosynthetic N relations (P_sat_–N and P_max_–N, V_cmax_–N, and J_max_–N) were all examined in relation to the factors affecting photosynthesis of a larch canopy. The goal of this study is to reveal seasonal and yearly variations in the photosynthetic capacity in situ of both short- and long-shoot needles in order to maintain the canopy function in a field.

## 2. Results

### 2.1. Seasonal Changes of Air Temperature and Soil Moisture

**Figure 1:**
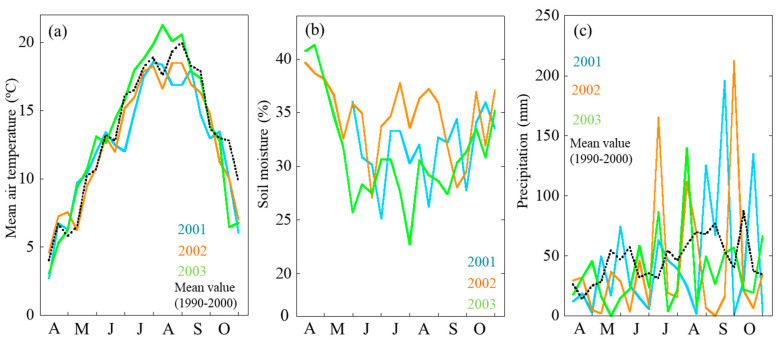
Seasonal and annual changes in mean daily air temperature (**a**), soil moisture (**b**) and precipitation (**c**). Different colors indicate the different year and mean value: light blue, 2001; orange, 2002; light green, 2003; dashed line, mean value from 1990 to 2000.

The peak air temperature was recorded in July and August. We should highlight that the air temperature in July and August 2001 and 2002 was much lower than that in 2003. In 2003, air temperature was higher, and soil moisture was recorded to be lower. From April to May, soil moisture in 2003 has sharply decreased as compared with that in 2001 and 2002. Soil moisture data during late April and May when short-shoot needles flushed indicated about 34% (*v*/*v*) in 2001 and 2002 while 27% in 2003. In late June to July, soil moisture was recorded to be at 35%, 31%, and 26% in 2001, 2002, and 2003, respectively (Figure 1).

### 2.2. Seasonal and Annual Changes in P_sat_, LMA, and Nitrogen Content

**Figure 2:**
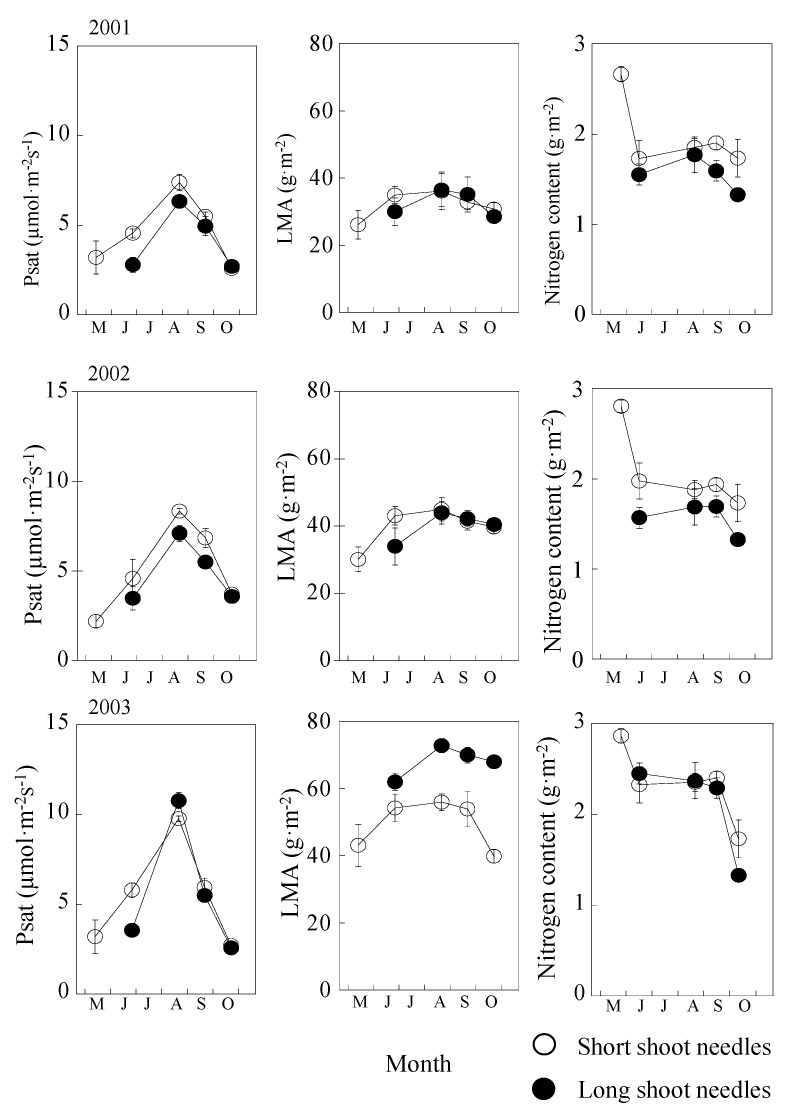
Seasonal and annual changes of P_sat_, LMA, and nitrogen content from 2001 to 2003. Open cycles are short shoots, and closed cycles are long shoots.

Short-shoot needles flushed in mid-May. After complete expansion of the short-shoot needles (about 20 days later), long-shoot needles started to develop. In short-shoot needles, P_sat_ gradually increased from mid-May to August and then decreased toward late October; the values ranged from about 2.0 to 8.0 µmol m^−2^s^−1^. Long-shoot needles displayed a similar tendency to short-shoot needles; however, P_sat_ of long-shoot needles increased from June to August and then decreased from late September toward October (Figure 2, left). Comparing the seasonal change in P_sat_, P_sat_ of short and long shoot was similar in 2001 and 2002. In contrast, there was a clear yearly difference in P_sat_ in 2003 (Figure 2, left). The P_sat_ of short shoots was generally higher than that of long shoots in 2001 and 2002, but a reverse trend was observed in 2003. Moreover, the difference between short and long shoots was significantly higher in the early phase of the growing season than in the late growing season (Figure 2, left). In the midst of summer, P_sat_ was found to be 30% higher in 2003 than in 2001 for short shoots and 40% higher for long shoots (Figure 2, left). However, the difference in P_sat_ between short and long shoots was found to insignificant (*p* > 0.05).

Except for the year 2003, no difference was found in the pattern of LMA of short- and long-shoot needles (Figure 2, center). LMA of both short- and long-shoot needles in 2003 showed 10–20 g m^−2^ higher than that in 2001 and 2002. After maturation of long-shoot needles in 2003, LMA of long-shoot needles did not clearly decrease, while LMA of both shoot needles slightly decreased in 2001 and 2002.

Leaf nitrogen (N) content of both types of needles showed similar pattern, in terms of seasonal change in the successive 3 years (Figure 2, right). N was high in May for short-shoot needles within the 3-year period. N showed a stable value with needle maturation in both needle types and then decreased after late September.

### 2.3. Photosynthesis–Nitrogen Relation

**Figure 3:**
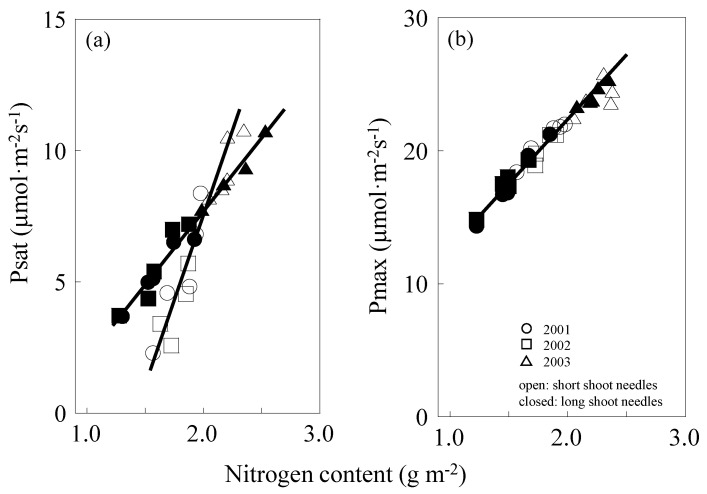
P_sat_–N relationship (**a**) and P_max_–N relationship (**b**). Open cycles are short shoots, and closed cycles are long shoots. Different shapes of symbols indicate different years, ○, 2001; □, 2002; △, 2003. Data are after leaf maturation till leaf senescence (June to October for short shoots, July to October for long shoots). Best-fitting equations: (**a**), P_sat_ = −3.90 + 5.62N, r^2^ = 0.97, *p* < 0.001 (long shoots); P_sat_ = −15.1 + 11.1N, r^2^ = 0.91, *p* < 0.001 (short shoots); (**b**), P_max_ = 2.22 + 9.47N, r^2^ = 0.90, *p* < 0.001 (short and long shoots).

Photosynthetic N use efficiency (PNUE) is expressed by the gradient of the linear part of the P_sat_–N relation. A steeper slope means more efficient use of N in photosynthesis. For both short and long shoots, accurate linear relations between leaf nitrogen and P_sat_ were observed. However, the difference between their gradients was found to be significant (*p* < 0.05). The gradient was steeper for short shoots (PNUE = 11.1) than for long shoots (PNUE = 5.6) (Figure 3a). PNUE of short-shoot needles was higher than that of the long-shoot needles. P_sat_ at CO_2_ and light saturation (=P_max_) and P_max_ of short and long shoots were equally correlated with their foliar N concentration (PNUE = 9.5) (Figure 3b).

### 2.4. Variation of V_cmax_ and J_max_

**Figure 4:**
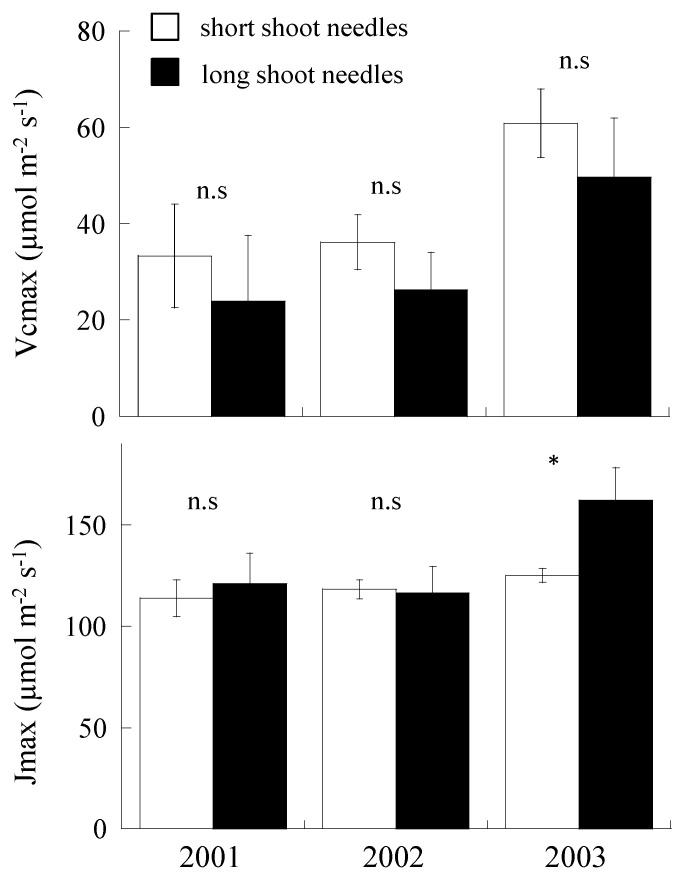
The maximum rates of RuBP carboxylation (V_cmax_) and the maximum rate of electron transport driving the RuBP regeneration (J_max_) of short- and long-shoot needles in August for 3 years. Open bars indicate short-shoot needles, while closed bars indicate long-shoot needles. n.s. means not significant. * indicates the statistical difference between short- and long-shoot needles. Vertical line on each bar indicates standard error (SE).

In V_cmax_, no significant difference was observed in V_cmax_ of short- and long-shoot needles (*p* > 0.05, Figure 4). However, in short-shoot needles, V_cmax_ of 2003 was significantly recorded to be higher than the other 2 years (*p* < 0.01). In long-shoot needles, V_cmax_ in 2003 tended to show marginally higher value than the other 2 years.

In J_max_, except 2003, no significant differences were observed in short- and long-shoot needles in 2001 and 2002. In 2003, J_max_ in short-shoot needles showed significantly lower value than that in long-shoot needles. In long-shoot needles, J_max_ value of 2003 was significantly higher than the other 2 years (*p* < 0.01).

### 2.5. V_cmax_, J_max_–N Relation

**Figure 5:**
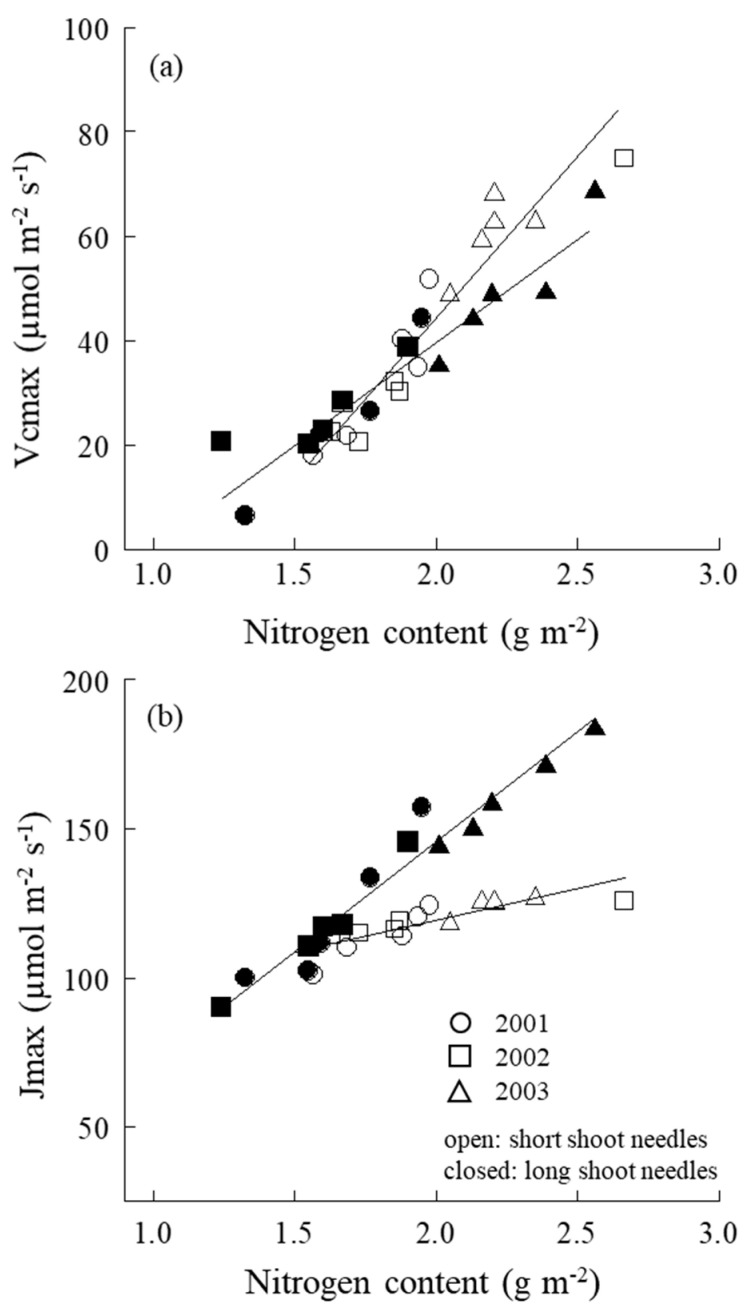
(**a**) Relationship between the maximum rates of RuBP carboxylation (V_cmax_) and leaf nitrogen content and (**b**) the maximum rate of electron transport driving the RuBP regeneration (J_max_) and leaf nitrogen content of both short- and long-shoot needles. ○, 2001; □, 2002; △, 2003. Open cycles are short-shoot needles, while closed cycles are long-shoot needles.

No marked difference was observed between short and long shoots in V_cmax_–N relation (Figure 5a). However, a significant difference between short-shoot and long-shoot needles in J_max_–N relation was also found, i.e., when N increased, J_max_ of short-shoot needles increased gentler than that of the long ones (Figure 5b).

### 2.6. Nitrogen Remobilization Rate (NRMR)

**Figure 6:**
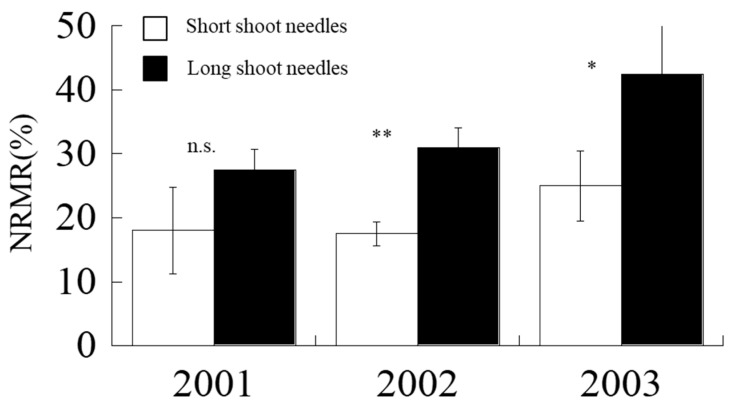
Nitrogen remobilization rates (NRMR) of short and long shoots from 2001 to 2003. Vertical line on each bar indicates standard error (SE). n.s. means not significant. * and ** imply significant at *p* < 0.05 and *p* < 0.01, respectively

Large annual variations in NRMR were observed for both short and long shoots (Figure 6). For short shoots, the minimum NRMR was recorded to be at 17%, observed in 2002, and the maximum NRMR was at 25% in 2003. For long shoots, the minimum NRMR was determined to be at 26%, and the maximum NRMR was at 43% (Figure 6). In each year, short shoots were able to remobilize less nitrogen during leaf senescence than that of long-shoot needles (*p* < 0.01) (Figure 6). On average, short shoots could remobilize 20% N, while long shoots could remobilize 33% N.

## 3. Discussion

### 3.1. Seasonal and Yearly Variation in Photosynthetic Rate: Climatic Limitation

Our study found that there were large seasonal and yearly variations in the photosynthetic capacity of the two shoot types (Figure 2). The pattern of seasonal changes in LMA and N was found to be similar in needles of both shoot types. As a result, a clear positive correlation was determined between N and P_sat_ or P_max_ (Figure 3). Photosynthetic capacity is determined both by climatic conditions and by status of leaf morphology, CO_2_ diffusion, N allocation to photosynthetic organs, kinetics of Rubisco, and others. [1,2,8,40,43,44]. In loblolly pine (*Pinus taeda*) canopy, year-to-year variation in its microenvironment led to large variations in P_sat_, which was shown as a stomatal limitation [34]. In a mixed deciduous forest, Bassow and Bazzaz [35] found that P_sat_ differs significantly not only between species but also among individuals within a species.

Temperature significantly affects the yearly variation of P_sat_ through optimal temperature for photosynthesis, vapor pressure deficit [1,2,32], and increases of leaf LMA and N [8,45,46]. The air temperature in July and August 2001 was at 3 °C lower than that in 2002 and 2003 and that in 2002 was about 1 °C lower than that in 2003 (Figure 1). There was a clear positive correlation between P_sat_ and N (Figure 3). From manipulated experiments in a greenhouse for four kinds of deciduous species with different leaf developments (determinant vs. indeterminant) [7], their P_sat_ was also found to positively correlate with LAM and N. With increasing temperature, larch needles were adequately developed with higher photosynthetic capacity. As a result, more energy from the photosynthates could be used in absorbing N; thus, more N was detected in needles (Figure 2).

Except in 2003, no significant difference was observed in LMA between short and long shoots (Figure 2) at leaf stable period [36], i.e., from the maturation of leaves to the start of leaf senescence. Significant high LMA in 2003 was observed in short-shoot leaves in June and in long-shoot leaves from July to September (Figure 2). In 2003, the soil moisture was much dryer than in 2001 and 2002 (Figure 1). Our finding of both shoots having higher LMA in the dry spring of 2003 suggests that the structure of needles was affected and changed by the low soil moisture conditions. Similar responses of the three kinds of understory seedlings in a Japanese larch forest to variations in climatic condition were reported in previous studies [1,8,43]. Soil moisture condition during leaf unfolding increased the P_sat_ of deciduous oak, magnolia, and hornbeam (*Carpinus* sp.) with high LMA and high N [8]. Similar responses were also reported. In the Central USA, maple seedlings had higher leaf mass leaves, responding to severe drought [6].

The evidence may indicate greater investment in leaf construction to endure desiccation in a water-limited environment. In dry and hot summer (2003), larch generally needs higher LMA, which is rewarded by higher photosynthetic rates per unit leaf area in both short and long shoots (Figure 2) and also higher NRMR for storage for the next year’s growth (Figure 6), which accord with the process of leaf economy [44,47]. Their findings [44,47] also agreed with our results. A previous study suggested that the different development stages of shoots and leaves may have important impacts on the seasonal course of photosynthesis [48]. This is one limitation of this study. The evaluation of combination effects of developing stage differences and impacts of climatic factors should be revealed in further studies.

### 3.2. Differences in the Photosynthetic Nitrogen Relations Between Short and Long Shoots

A positive correlation between leaf N and photosynthesis has been observed across many species [1,2,49] with large variations among species [47], even in yearly variation within the same species [28]. Weak linear relations have also been observed in some canopy species [50]. This disparity is considered to be attributed to the fact that photosynthetic capacity rises linearly with increasing N until limitation of other factors [1,2]. Although accurate linear relations of P_sat_–N in both short and long shoots were recorded, this difference became negligible when the P_max_ took place of P_sat_, indicating that CO_2_ diffusion may be a reason for this disparity (Figure 3) We should know which photosynthetic processes are most affected by this CO_2_ diffusion differences, such as mesophyll conductance or cell wall resistance [40,41].

V_cmax_ linearly increased with leaf N, and no differences in V_cmax_–N were found between short and long shoots (Figure 5a). On the other hand, J_max_ of long-shoot needles increased steeper with an increase in leaf N compared with that of the short ones (Figure 5b). Thus, although the efficiency of leaf N in carboxylation enzymes of short and long shoots was quite similar, the less efficiently in RuBP regeneration for short-shoot needles decreased the J_max_, finally resulting in the apparent differences observed in P_sat_–N (Figure 3a). Surely, the diffusion difference between short and long shoots strongly affects the N use in RuBP regeneration, but not the carboxylation processes.

Given that N could be equally allocated between carboxylation and RuBP regeneration, one possible reason for the less N efficiency in RuBP regeneration may be attributed to the shortage of other resources, such as CO_2_. It has been proved that P_sat_ at CO_2_ and light saturation (=P_max_) may indicate maximum photosynthetic without any limitation of stomata; therefore, there should be clear positive correlation between N and P_max_ (Figure 3b).

We found that N remobilized at different rates in short and long shoots; for instance, short shoots tended to remobilize less N during leaf senescence compared with long shoots (Figure 6), especially in 2003 when we had relatively high temperature and dry spring (Figure 1). Moreover, long-shoot needles are positioned at the branch top [17], where elongation in the next year will occur. Therefore, we considered that this process is essential in the development of long shoot as it can store more nutrients for the next year’s growth at the shoot top [29].

LMA stands for leaf level function with changes in N allocation to photosynthetic protein [9,51,52]. Therefore, yearly variation of LMA, mainly attributed to temperature and humidity changes, could affect the annual variation of PNUE and P_sat_.

The photosynthesis–N relation of larch needles with different shoot types is considered to be surely affected by several factors [53]: CO_2_ diffusion, leaf N, stomatal conductance, and kinetics of Rubisco (Figure 3). Hikosaka et al. [54] also suggested that PNUE differences between evergreen broadleaved trees and annual herbaceous plants may be caused not by a single factor but a combination of several factors. Their conclusion agrees with our findings, i.e., yearly variation of P_sat_–N and P_sat_–N can be induced by a combination of changes brought about by LMA, N content, N allocated to photosynthetic protein [49,50,53,54,55,56,57], etc. in the needles.

In conclusion, as a typical heterophyllous conifer, Japanese larch trees display no potential difference in photosynthetic rate (P_sat_) between short and long shoots, which only shows that climate factors, such as high temperature and soil moisture during leaf development rather than shoot types, affect the P_sat_. However, the P_sat_–N of short- and long-shoot needles was different, although this difference was reduced under conditions of saturation with CO_2_ and light (i.e., P_max_–N). No differences were observed in V_cmax_–N; however, J_max_–N showed that J_max_ of short-shoot needles was suppressed, and this trend was presented as a gentler slope than that of the long ones. Moreover, much higher N remobilization rates were found in long shoots for the successive growth the following year.

Therefore, climate factors can affect the morphological (LMA) and physiological traits (N, V_cmax_, J_max_) of shoot needles [3,38,48,58,59,60,61]; thus, a combination of these external and internal factors can result in the yearly variation of P_sat_ and P_sat_–N relationship in short- and long-shoot needles. With the changing climate, Japanese larch trees would be able to cope with high temperature and drought to some extent via high plasticity in LMA of both short- and long-shoot needles.

## 4. Materials and Methods

### 4.1. Study Site

The study was conducted in a larch plantation (50 years old as of 2003) located at Tomakomai National Forest, northern Japan (42°40′ N, 141°36′ E, 200–300 m a.s.l.) from 2001 to 2003. The soil is comprised of immature volcanic ash (vitric andosols) and is very shallow (around 15–20 cm), which is an aftereffect of the eruption of Mt. Tarumae in 1739. The mean annual precipitation is about 1250 mm, and the mean monthly air temperature ranges from −3.2 °C to 19.1 °C. Typical rainy season is from mid-July to September. Typical freezing season is from December to March, and the coldest season is from late January to early February.

### 4.2. Plant Materials

A 20 m tower with walkway was built to reach the shady and sunny crown of the three mature trees of larch (*Larix kaempferi* (Lamb.) *Carrière*) with *heterophyllous* shoots (48 years old as of 2001). From late April to early May, buds of short-shoot needles flushed, and 2–3 weeks later, long-shoot needles started succeeding growth. On late August, the growth of long-shoot needles stopped. At late September to early October, both short- and long-shoot needles start to color, and by mid to late October, larch trees started shedding of their needles. As shown in the previous studies [16,29], separated needles of long shoots grow directly on current-year branches, and these needles are found to be generally thick and had a large connection interface between leaves and branches. Meanwhile, short shoots grow on older branches with bundles of needle leaves, and these needles generally had a thin leaf blade and small connection interface [30,31]. The average height of the larch plantation was 12.3 m. From 2001 to 2003, three replications in each for short and long shoots from the upper canopy of three individual trees were selected for the measurement. For 1 year, we measured the photosynthetic rates of the short-shoot needles five times (a total of 15 shoots was used as sample) and of the long-shoot needles four times (a total of 12 shoots). The total number of short and long shoots was 45 and 36, respectively, for 3 years.

### 4.3. Air Temperature and Soil Moisture Monitoring

Temperatures at the study site were monitored through auto-logged climatic monitors (HMP45D, VAISALA, Helsinki, Finland) at a height of 8 m aboveground. The soil moisture was measured by the TDR sensor (CS615, Campbell Scientific Inc., Logan, UT, USA) at a depth of 0.2 m. The data were recorded twice per hour (Asia Flux web: http://asiaflux.net/index.php?page_id=113).

### 4.4. Photosynthesis and Nitrogen Measurement of Needles

All of the gas exchanges of short and long shoots were measured using a portable gas analyzer LI-6400 (LI-Cor, Lincoln, NE, USA) with transparent conifer chamber (6400-05), equipped with a 2050 HB illumination system (Walz, Effeltrich, Germany) to determine light-saturated photosynthetic rates at 360 ppm CO_2_ (P_sat_). Today, CO_2_ concentrations reach to 410 ppm, and 50 ppm differences are present; however we think that the effect of the differences to photosynthetic rates would be minor, based on the results of photosynthetic responses of larch species to the elevated CO_2_ [58]. Approximately 5 cm shoots enclosed the chamber. Measurements of P_sat_ were made under steady-state conditions at a leaf temperature of 24 –28 °C and the photosynthetic photon flux density (PPFD, µmol m^−2^s^−1^) of about 2000 µmol m^−2^s^−1^. After determining P_sat_, we calculated the P_max_ by increasing the CO_2_ concentration in increments of 100 ppm up to 1500–1800 ppm. Stomatal conductance was maintained above 0.05 mol m^−2^s^−1^ during the measurement of P_max_.

After the each gas exchange measurement, the measured shoots were harvested and brought back to the laboratory. The leaf area (A) was then measured using a LI-3000 leaf area system (LI-Cor); the result was used to calculate the photosynthetic rate per unit area by recalculation program of LI-6400. Following this area determination, the needle dry mass (M) was measured at 65 °C for 48 h, and the LMA was calculated according to Pérez-Harguindeguy et al. [59], namely:

LMA = M/A (1)

Foliar nitrogen content (N) was determined using an NC analyzer (NC-900, Shimadzu, Kyoto, Japan). The number of needles ranged between six and ten for each time. The N was calibrated and checked against a standard (acetanilide: N = 10.36%, C = 71.09%; Wako, Osaka, Japan).

### 4.5. Analysis of A-Ci Curves

A-Ci curves can be used to estimate V_cmax_ (maximum rate of carboxylation allowed by Rubisco), and J_max_ (maximum electron transportation rates) was estimated by the model [42,43,51] displayed as follows:

P_n_ = min (W_c_, W_j_) (2)

where W_c_ is the Rubisco-limited net photosynthetic rate (µmol m^−2^ s^−1^) and W_j_ is the RuBP regeneration rate-limited net photosynthetic rate (µmol m^−2^ s^−1^). The W_c_ and W_j_ are provided by Equations (3) and (4):

(3) $$W_{c}=\frac{V_{\mathrm{cmax}}\;\left(C_{c}-\Gamma^{*}\right)}{C_{i}+K_{c}\left(1+O/K_{o}\right)}-R_{d\;}$$

(4) $$W_{j}\;=\;\frac{J\;\left(C_{i}-\Gamma^{*}\right)}{{4C}_{i}+8\Gamma^{*}}-R_{d}$$

In Equation (4) at light saturation, J is equal to J_max_ [42]; therefore:

(5) $$W_{j}\;=\;\frac{J_{\max}\;\left(C_{i}-\Gamma^{*}\right)}{{4C}_{i}+8\Gamma^{*}}-R_{d}$$

Here, P_n_ is the net photosynthetic rate, V_cmax_ is the maximum rate of carboxylation allowed by Rubisco, J is the potential electron transport rate, Ci is the intercellular concentration of CO_2_ (µmol m^−2^s^−1^) and O that of O_2_, K_c_ is the Michaelis–Menten constant for carboxylation and K_o_ that for oxygenation, Γ* is the CO_2_ compensation point (µmol m^−2^s^−1^), R_d_ denotes day respiration (µmol m^−2^ s^−1^), W_c_ is the Rubisco-limited rate (potential rate limited by the activity of Rubisco and the concentration of CO_2_ and O_2_), W_j_ is RuBP-limited rates (the rate of RuBP carboxylation in photosynthesis is either equal to the potential rate allowed by the concentration of RuBP), and Γ* = 3.69 (KPa), Kc = 40.4 (KPa), Ko = 24.8 (KPa) [42,56]. In this study, we calculated the apparent V_cmax_ and J_max_ because we did not estimate mesophyll conductance for CO_2_ diffusion, the same as the other studies [48,49].

### 4.6. Calculation of Nitrogen Remobilization During Autumn Senescence

Several studies have examined N remobilization from aged leaves [8,29,59,60,61]. We express the N remobilization rate (NRMR) as:

(6) $$\mathrm{NRMR}\left(\%\right)=\left(\frac{N_{\mathrm{LSP}}\;-{\;N}_{\mathrm{died}}}{N_{\mathrm{LSP}}}\right)\;\times 100$$

where N_LSP_ is the mean value of N (g m^−2^) in leaf stable period (LSP, [36]) and N_died_ is the N content of leaves (g m^−2^) collected in late October, just before leaf shedding [8].

### 4.7. Statistical Analysis

All statistical tests were carried out using the R language (R developing core team, Vienna). To compare the P_sat_–N linear relation, the V_cmax_–linear relation, and J_max_–N linear relation, we used analysis of covariance (ANCOVA). To compare the P_sat_ for long and short shoots, we use *t*-tests. To compare the seasonal and yearly fluctuations in photosynthesis and leaf characteristics, and to compare the difference in photosynthetic capacity and the NRMR between short and long shoots, we used component analysis of variance.
